# Unlike for Human Monocytes after LPS Activation, Release of TNF-α by THP-1 Cells Is Produced by a TACE Catalytically Different from Constitutive TACE

**DOI:** 10.1371/journal.pone.0034184

**Published:** 2012-03-30

**Authors:** Helena Moreira-Tabaka, Jean Peluso, Jean-Luc Vonesch, Didier Hentsch, Pascal Kessler, Jean-Marie Reimund, Serge Dumont, Christian D. Muller

**Affiliations:** 1 Laboratoire d'Innovation Thérapeutique, UMR 7200, Faculté de Pharmacie, Université de Strasbourg, Illkirch, France; 2 Centre d'Imagerie, Institut de Génétique et de Biologie Moléculaire et Cellulaire, INSERM U596, CNRS UMR 7104, Université de Strasbourg, Illkirch, France; 3 CHU de Caen, Service d'Hépato-Gastro-Entérologie et Nutrition, Pôle Médecine d'Organes et Cancérologie, Caen, France; 4 Université de Caen Basse-Normandie, Laboratoire Microenvironnement Cellulaire et Pathologies, UFR de Médecine, CHU de Caen, Caen, France; University of North Dakota, United States of America

## Abstract

**Background:**

Tumor necrosis factor-alpha (TNF-α) is a pro-inflammatory cytokine today identified as a key mediator of several chronic inflammatory diseases. TNF-α, initially synthesized as a membrane-anchored precursor (pro-TNF-α), is processed by proteolytic cleavage to generate the secreted mature form. TNF-α converting enzyme (TACE) is currently the first and single protease described as responsible for the inducible release of soluble TNF-α.

**Methodology/Principal Findings:**

Here, we demonstrated the presence on THP-1 cells as on human monocytes of a constitutive proteolytical activity able to cleave pro-TNF-α. Revelation of the cell surface TACE protein expression confirmed that the observed catalytic activity is due to TACE. However, further studies using effective and innovative TNF-α inhibitors, as well as a highly selective TACE inhibitor, support the presence of a catalytically different sheddase activity on LPS activated THP-1 cells. It appears that this catalytically different TACE protease activity might have a significant contribution to TNF-α release in LPS activated THP-1 cells, by contrast to human monocytes where the TACE activity remains catalytically unchanged even after LPS activation.

**Conclusions/Significance:**

On the surface of LPS activated THP-1 cells we identified a releasing TNF-α activity, catalytically different from the sheddase activity observed on human monocytes from healthy donors. This catalytically-modified TACE activity is different from the constitutive shedding activity and appears only upon stimulation by LPS.

## Introduction

Tumor Necrosis Factor-α (TNF-α) is a pro-inflammatory cytokine produced and secreted primarily by macrophages and monocytes in response to a bacterial challenge or tumor burden. Tumor Necrosis Factor-α plays a key role in host defense and immunosurveillance. Overproduction and secretion of TNF-α may have detrimental effects and are strongly involved in acute inflammation, in chronic inflammatory diseases, and probably also in several cancer types initiation (e.g. colorectal cancer complicating colonic inflammatory bowel diseases).

Large amounts of TNF-α are released in response to lipopolysaccharide (LPS) and other bacterial products (e.g. staphylococcal enterotoxin B, bacterial super antigen toxin 1). However, several other agents can also modulate its expression including other cytokines such as interleukin 1 and 2 (IL-1 and IL-2), TNF-α itself, cross-linking FcγR, granulocyte-monocyte colony-stimulating-factor (GM-CSF), macrophage colony-stimulating-factor (M-CSF), CD40 ligand, glucocorticoids and phorbol esters [1], [2], [3], [4]. It is well established that the induction of TNF-α production upon stimulation by LPS results from both an enhancement of TNF-α gene transcription and a translational derepression of its mRNA. In non-stimulated macrophages, TNF- α m-RNA translation is blocked. Upon activation by LPS this repression is overcome. The newly synthesized TNF-α protein initially accumulates primarily within the Golgi complex and then goes through the secretory pathway up to the cell surface [3]. The TNF-α is transported from the trans-Golgi network (TGN) in tubular carriers/vesicles to the recycling endosome (RE), which serve as an intermediary compartment before cell surface delivery [5].

A transient appearance of TNF-α on the cell plasma membrane is followed by degradation/endocytosis [6] or by proteolytical cleavage of the full length TNF-α transmembrane protein (pro-TNF-α or m-TNF-α) to release a soluble mature form (s-TNF-α) [7]. This process called “ectodomain shedding” remains a critical point regulating TNF-α secretion and function, providing a strong incentive to define the responsible sheddases and to understand the underlying proteolytic machinery. Previous studies demonstrated that pro-TNF-α can be processed by a membrane associated serine protease or metalloprotease, and that metalloprotease inhibitors can effectively inhibit the release of s-TNF-α [8], [9], [10]. Up to now, only one enzyme - TACE/ADAM17 (Tumor Necrosis Factor Converting Enzyme, EC 3.4.24.86) - has been found to specifically cleave pro-TNF-α [11], [12], despite the constitutive shedding of TNF-α seems not to be managed only by TACE [13], [14], [15], [16], [17]. Beside TACE, several other ADAMs have been implicated in TNF-α shedding, i.e. ADAM10, most closely related to TACE [18], [19], as well as ADAMs 9 and 19 [17], [20]. Whilst ADAM10 and TACE can cleave a peptide mimicking the physiological cleavage site of TNF-α, in exactly the same position that in cells (SPLAQA76-V77RSSSR), the others (ADAM9 and 19) do not match the physiologically relevant site (cleavage sites are: SPLA-QAVRSSSR for ADAM 9 and SPLAQAVRS-SSR and SPLAQAVR-SSSR for ADAM19). Other enzymes have been considered to be implicated as well in TNF-α shedding, including MMP7/matrilysin [21] and serine protease PR3 (cleavage site: SPLAQAV-RSSSR) [7], [9]. Taking all together, there is some evidence for multiple sheddase activities being involved in the cleavage of TNF-α. Nevertheless, the mechanism of in vivo regulation of shedding activity is not yet well understood. One can hypothesize that these activities may differ depending on several factors such as cell types, constitutive or induction conditions and acute or chronic inflammatory conditions. Therefore, this work was designed to provide a better understanding of the molecular phenomenon's associated with TNF-α release from secreting cells. For this purpose, we used three original TNF-α secretion inhibitors: Thymoquinone (TQ), Celastrol and Ro 32-7315. Thymoquinone and Celastrol are synthetic molecules derived from plants. Thymoquinone is the main active constituent of the volatile oil of *Nigella sativa* black seeds [22], whereas Celastrol is a dienone-phenolic triterpen isolated from *Tripterygium wilfordii* [23]. Both compounds have been previously described as potent anti-oxidant, and anti-inflammatory molecules, able to inhibit TNF-α production [24], [25], [26]. Ro 32-7315 was developed by ROCHE as a potent, orally active TACE inhibitor, based on succinate hydroxamate scaffold, which reached phase I clinical trials [27], [28]. Ro 32-7315 displays 100–500-fold selectivity over MMPs-1,-2,-3,-9,-13 but not for MMP-8 [29], [30].

## Results

### The specific TACE inhibitor Ro 32-7315, TQ and Celastrol confirmed a high potency to inhibit TNF-α secretion

**Figure 1:**
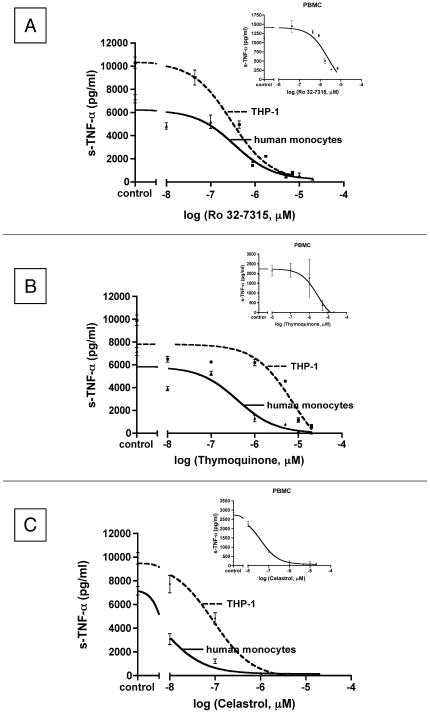
Inhibition of TNF-α secretion by different human cell types in presence of Ro 32-7315, TQ and Celastrol. Cells (5×10^5^/ml) were incubated for 4 hours (for human monocytes and THP-1 cells) or 24 hours (for PBMC) with 1or 5 µg/ml LPS respectively, in the presence of ranging compounds concentrations or 0.1% (v/v) DMSO as a control. The TNF-α levels were then assessed in culture supernatant by ELISA technique. Dose-response curves were fitted on GraphPad PRISM (v.4.0, GraphPad Software, Inc. La Jolla, CA,USA) sigmoidal dose-response curve-fit model (n = 3 independent experiments); s-TNF-α correspond to the secreted form of TNF-α. Statistical difference between IC50 of Ro 32-7315, TQ and Celastrol are indicated in Table 1.

Ro 32-7315, TQ and Celastrol have been selected here as well characterized anti-TNF-α compounds. First we aimed to assess their anti-TNF-α potency in our cell-based inflammation models: human mononuclear cells isolated from peripheral blood (PBMCs), human monocytes purified from peripheral blood (PBMs) and the human monocytic cell line (THP-1). In order to obtain a TNF-α secretion mimicking tissue inflammatory activation, cells were activated by lipopolysaccharide (LPS), a major inducer of TNF-α (production and release). The LPS dose respectively used for each cell type (PBMC 5 µg/ml; THP-1 1 µg/ml) is issued from a “data not shown” dose response. Inhibition of LPS induced TNF-α release by Ro 32-7315, TQ and Celastrol, was measured for different concentrations. As illustrated in Figure 1a, Ro 32-7315 induced a dose dependent inhibition of TNF-α release and this for all the 3 tested cell types. Concentrations lower than 10 µM were sufficient to obtain a complete inhibition of cytokine secretion. In a similar way, both TQ and Celastrol inhibited TNF-α secretion in a dose-dependent manner and were able to completely block its secretion (Figure 1b and 1c). In our hands, Celastrol presented the best anti-TNF-α activity, much better than the specific TACE inhibitor, Ro 32-7315.

In the range of tested concentrations, cell viability was not affected for all tested compounds (data not shown), confirming that the observed effects were due to their anti-TNF-α activity and not to cell death. Their strong potency to block TNF-α release is highlighted by their low IC50 values as shown in Table 1.

**Table 1:** IC_50_ (µM) values of tested compounds for TNF-α release by human monocytes, THP-1 cells and PBMC (n = 3 independent experiments) are estimated using GraphPad PRISM® 5.0 d (GraphPad Software, Inc., San Diego CA).

Compounds	IC50 (nM)		
	THP-1^A^	Monocytes^B^	PBMC^C^
Ro 32-7315	290±132	360±48	2400±175
Thymoquinone	7400±1885	4000±192	2200±174
Celastrol	90±22	0.46±0.1	44±17

The half-maximal inhibition values (IC_50_) for each tested compounds is determined by fitting the data to the sigmoid dose-response equation:
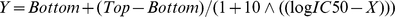
where X is the logarithm of the compound's concentration, Y is the TNF-**α** release. Bottom the baseline response and Top is the maximum response.

*Statistical significances:* (**A**) IC50's in THP-1 are significantly different between the 3 compounds; p = 0.027 (Kruskal-Wallis). As a result we could compare each compound individually to the two others (Mann Whitney): Ro-32-7315 vs. Thymoquinone, p = 0.049; Ro-32-7315 vs. Celastrol, p = 0.049; Thymoquinone vs. Celastrol, p = 0.049; (**B**) same statistical significances in monocytes than in THP-1 cells A. (**C**) p = 0,039 for PBMC (Kruskall-Wallis); Ro-32-7315 vs. Thymoquinone, p>0.05 (not significant); Ro-32-7315 vs. Celastrol, p = 0.049; Thymoquinone vs. Celastrol, p = 0.049.

### TNF-α accumulation onto cell surface after specific TACE inhibition

**Figure 2:**
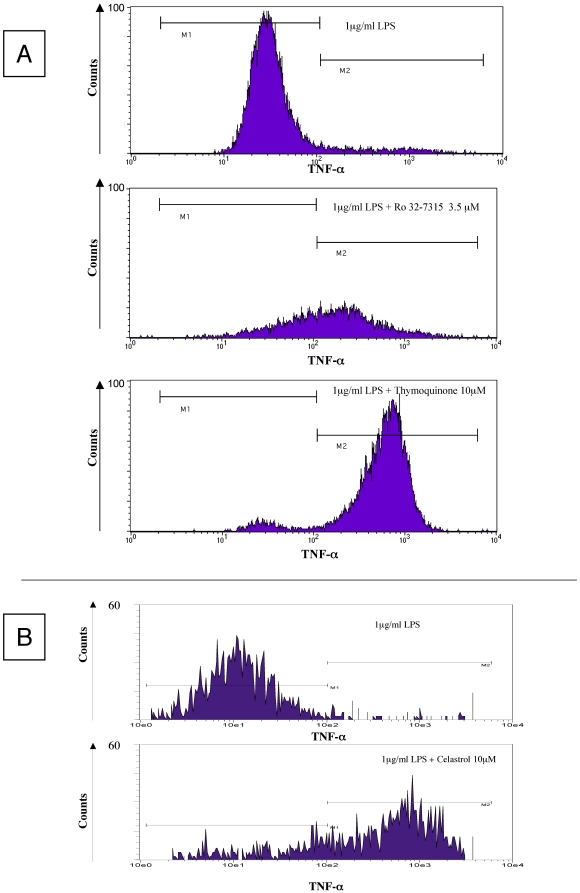
THP-1 cell surface accumulation of membrane-bound TNF-α after Ro 32-7315 and TQ (Panel A) or Celastrol (Panel B) treatment. Cells were stimulated for 4 hours (1 µg/ml of LPS) in the presence of Ro 32-7315 (3.5 µM), TQ (10 µM) and Celastrol (10 µM) or 0.1% (v/v) DMSO as a control. The intact cells were then stained by FITC-anti-m-TNF-α MAb. Membrane-bound TNF-α expression was evaluated in flow cytometry. Representative histograms are shown. The fluorescence intensity (AU) is plotted versus the number of cells. For all 3 compounds, membrane –bound TNF-α expression increased after THP-1 cells were treated (p<0.05).

**Figure 3:**
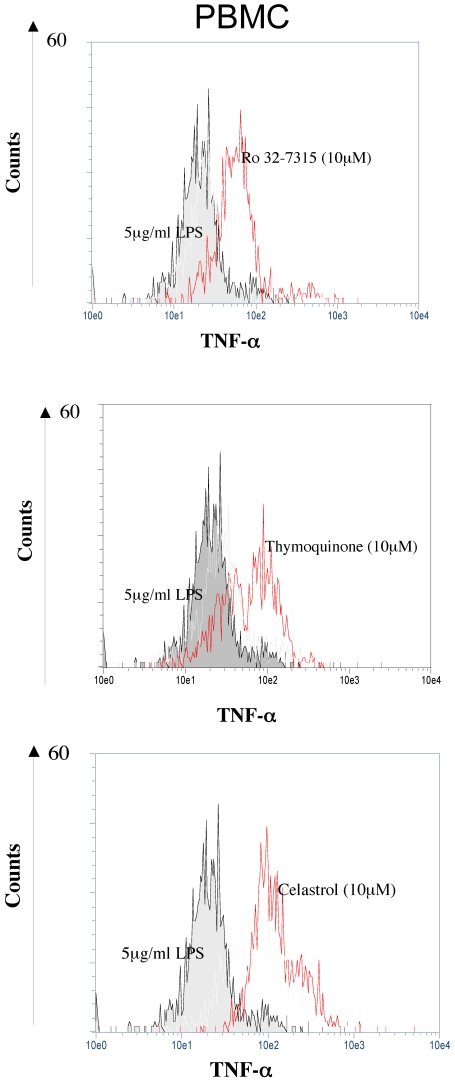
PBMC surface accumulation of the membrane-bound TNF-α after Ro 32-7315, Celastrol and TQ treatment. Cells were stimulated for 24 hours with 5 µg/ml of LPS in the presence of 10 µM of Ro 32-7315, TQ and Celastrol. 0.1% (v/v) DMSO was used as a control (silver histogram). The non-permeabilized cells were then stained with FITC-anti-m-TNF-α MAb. Membrane TNF-α expression was evaluated by flow cytometry. The representative histograms are shown. The fluorescence intensity (AU) is plotted against the number of cells (y-axis). For all 3 compounds, membrane–bound TNF-α expression increased after THP-1 cells were treated (p<0.05, n = 3).

**Figure 4:**
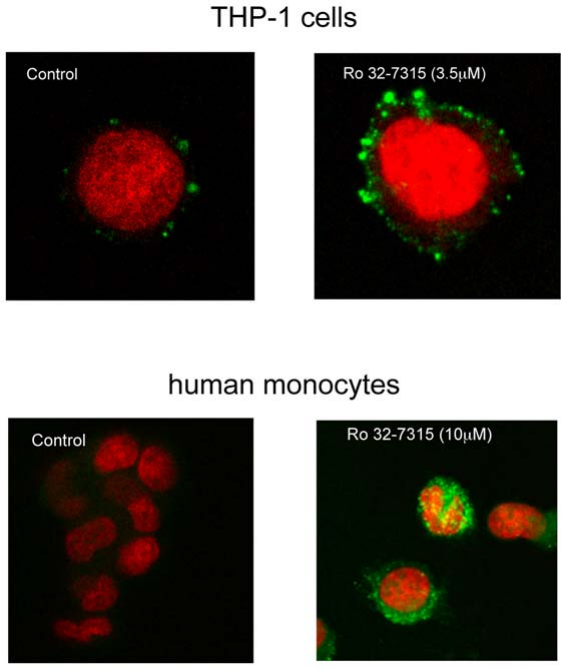
Immunofluorescence of membrane-bound TNF-α on the surface of THP-1 cells and human monocytes after Ro 32-7315 treatment. Cells were stimulated for 4 hours with LPS (1 µg/ml) in the presence of Ro 32-7315. Then, cells were stained by FITC-anti-m-TNF-α MAb. Image acquisition was made on a confocal microscope. The numerical recording of the images and analysis were carried out in Image J (Wayne Rasband, National Institute of Mental Health, Bethesda, Maryland, USA.).

TNF-α is initially synthesized as a transmembrane precursor (m-TNF-α), then processed by TACE and/or other sheddase to become a secreted soluble form. Blocking of this process e.g. by TACE inhibitors should thus generate TNF-α retention and accumulation on the cellular membrane. To verify the cogency of our hypothesis, we evaluated m-TNF-α expression in cells pretreated by Ro 32-7315, a specific TACE inhibitor. As demonstrated by flow cytometry (Figure 2 panel A and Figure 3), Ro 32-7315 treatment yielded in an important accumulation of m-TNF-α. The obtained MFI (AU) values for THP-1 cells and PBMC were 260 vs. 64 (p = 0.049) and 90 vs. 30 (p = 0.046) (treated vs. control cells), respectively. We further performed confocal microscopy in order to validate the membrane localization of retained TNF-α after TACE inhibitor treatment. As shown in Figure 4, a strong cell surface staining was visible in the presence of Ro 32-7315 in both THP-1 and monocytes. Visualization of membrane anchored TNF-α revealed a punctuated distribution at the cell surface, suggesting a lipid raft localization of TNF-α. Tellier *et al.* have shown that the mature form of TNF-α is mainly present in the cholesterol-rich membrane microdomains (lipid rafts) and that an inhibition of metalloproteases increases the proportion of TNF-α in those parts [31]. Thus our results confirm that inhibiting TACE activity leads to an accumulation of unprocessed TNF-α on the cell surface.

### Thymoquinone and Celastrol induce an accumulation of cell surface m-TNF-α

As for Ro 32-7315, we demonstrated that TQ and Celastrol, are both able to produce a substantial increase in the m-TNF-α expression (Figure 2 panel A and B and Figure 3). The MFI (AU) values obtained for those agents were: 635 vs 64 (THP-1 cells; p = 0.049) and 81 vs 30 (PBMC; p = 0.046) for TQ; 663 vs 86 (THP-1; p = 0.049) and 170 vs 30 (PBMC; p = 0.049) for Celastrol. The observed properties of TQ and Celastrol to strongly inhibit TNF-α secretion together with important capacity to retain uncleaved TNF-α on the cell surface indicate that both are able to block m-TNF-α processing strongly suggesting an anti-TACE activity.

### Cell surface TACE expression on THP-1 cells and PBMCs

**Figure 5:**
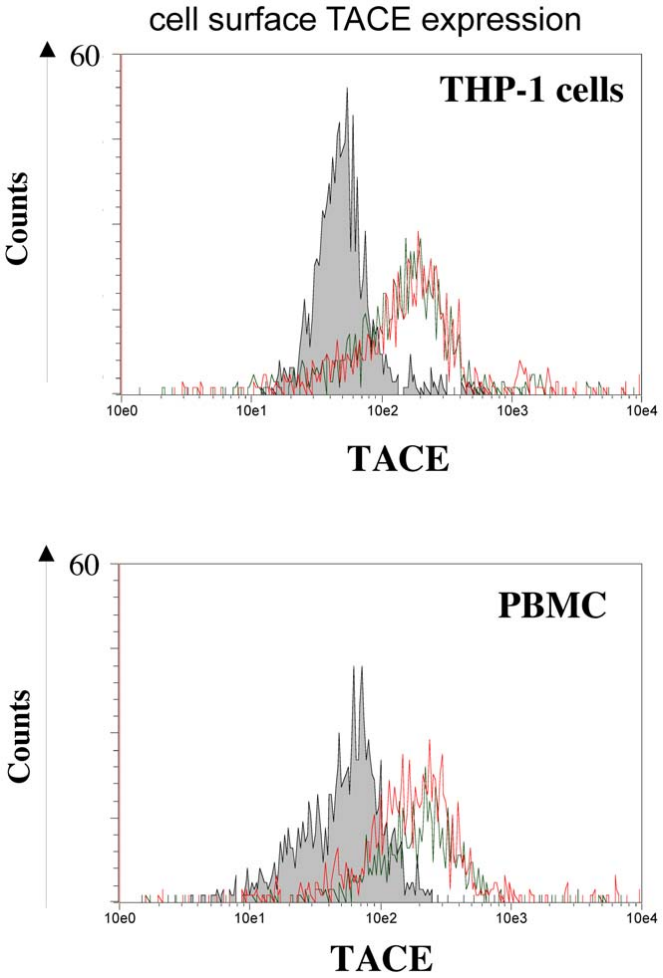
TACE expression on surface of THP-1 Cells and PBMCs. Non-activated or LPS stimulated cells (4 hours by 1 µg/ml of LPS for THP-1 cells or 24 hours by 5 µg/ml of LPS for PBMC) were stained with Phycoerythrin labelled anti-TACE MAb. Cell surface TACE expression was then evaluated by flow cytometric analysis. The ordinate relates to the relevant cell number, while fluorescence intensity can bee seen on the abscissa, representing cell surface TACE level. Silver histogram show unstained cells. Green and red lines present stained cells, non-activated and activated, respectively. Difference is statistically significant (p = 0.049 for both THP-1 and PBMC, n = 3).

To provide evidence for TACE expression on THP-1 cells as well as on PBMCs, we performed flow cytometry analysis of LPS-activated and non-activated cells (Figure 5). Both THP-1 and PBMC resting cells demonstrated a cell surface TACE expression with MFI values (AU) of 270 and 300 respectively, compared to 70 (p = 0.049) and 66 (p = 0.049) for unstained cells. Treatment of the THP-1 cells and PBMC by LPS for 4 or 24 hours respectively, corresponding to the maximum release of soluble TNF-α secretion accordingly to each cell type, did not change significantly the levels of TACE protein expression (MFI values: 290 AU for THP-1 cells and 320 AU for PBMC, p = 0.657), indicating that TACE expression in both cell types does not depend from LPS stimulation. These data are consistent with those described in the literature, where no increase in the amount of surface TACE has been found in response to various cell activators [32]. Moreover, Doedens and Black demonstrated that LPS stimulation of THP-1 cells has no effect on TACE localization or expression suggesting that the basal level of TACE activity may be sufficient for the release of TNF-α from LPS stimulated monocytic cells. Thus the effect of LPS may be solely to induce neo-synthesis of TNF-α, which is then released after constitutive TACE activity [33]. Another explanation, suggested by Li et al., could be that TACE molecular structure changes or is degraded after TNF-α secretion [14].

### THP-1 cells and PBMs surface associated enzymatic activity

After having shown the presence of TACE, we analyzed in a next step TACE enzymatic activities present on THP-1 cells and PBMs. For this purpose a highly sensitive fluorometric assay based on fluorescence-quenched (FRET) peptides as substrates was used. This method permitted a continuous real-time monitoring of the enzyme activity on the surface of living cells. Two FRET substrates have been considered of interest: i) the first is MOCA, a broad-spectrum MMP substrate, particularly sensitive for testing collagenase (MMP-1, -8, 13), TACE and ADAM 10, ii) the second is ABZ, a highly TACE specific substrate.

Intact THP-1 cells, as PBMs, were found able to hydrolyze each peptide, indicating the presence of constitutive enzymatic activities on the cell surface (data not shown).

**Figure 6:**
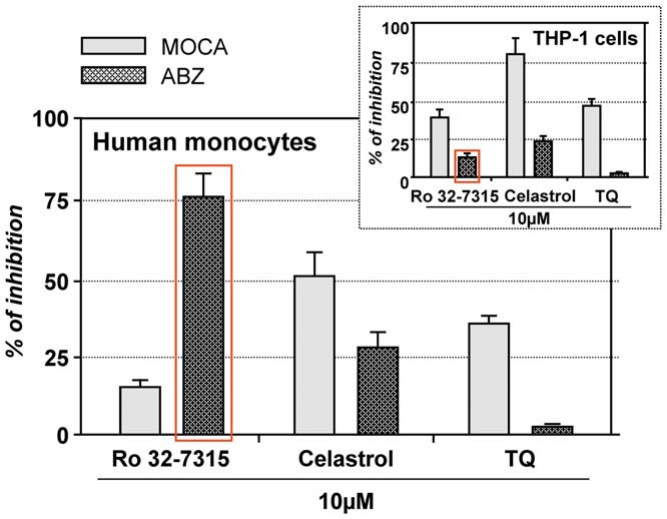
Effects of Ro 32-7315, Celastrol and TQ on MOCA and ABZ substrates hydrolysis by human monocytes as compared to the THP-1 cell line (inset). The enzymatic activity was determined for viable intact cells using MOCA and ABZ substrates (n = 3). Thus, 0.5×10^6^/ml of viable cells were incubated with 5 µM of the substrate in Ca^2+^ and Mg^2+^ free HBSS in the presence of compounds (10 µM). Fluorescence intensity was monitored for 600 s, and the rates of the peptide hydrolysis were then calculated from the linear section of the fluorescence curve. The inhibitory potency of the compounds was calculated by divining the reaction rates of substrate hydrolysis obtained for different compounds concentrations **(V(substrate)_[inhibitor]_)** by substrate hydrolysis reaction rate with DMSO addition (**V(substrate)_[0.1% DMSO]_**). Inhibitory potency (%) = 100%−(V(substrate)[inhibitor]/V(substrate)[0.1% DMSO])×100%.

**Figure 7:**
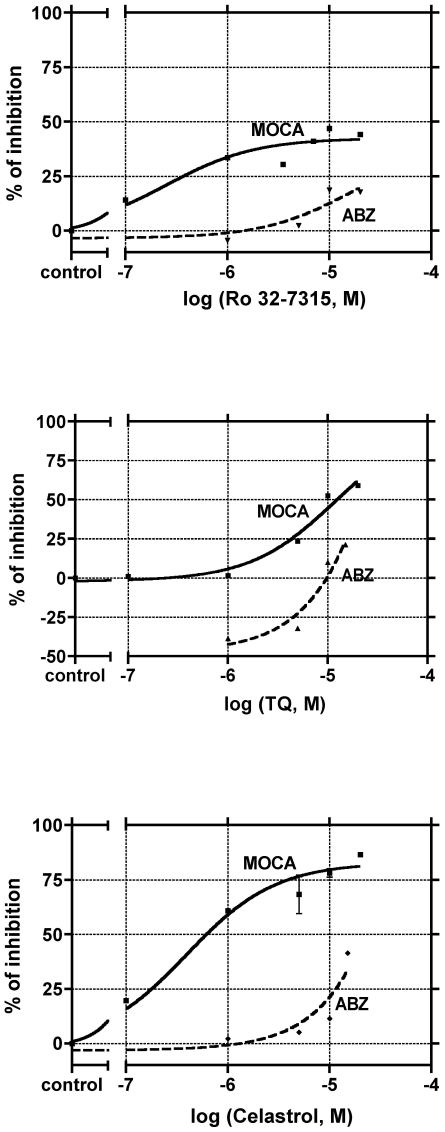
Effects of Ro 32-7315, Celastrol and TQ on MOCA and ABZ substrates hydrolysis by THP-1 cells. The viable intact THP-1 cells (0.5×10^6^ cells/ml) were incubated with 5 µM of the MOCA or ABZ substrate in Ca^2+^ and Mg^2+^ free HBSS in the presence of various concentrations of Ro 32-7315, Celastrol or TQ. The final DMSO concentration in the reaction mixture was 0.1%. The fluorescence intensity was monitored for 600 s and the rates of the substrates hydrolysis were calculated from the linear section of the fluorescence curve. To estimate the accurate inhibitory potency of the tested compounds, the reaction rates of MOCA hydrolysis obtained for different compounds concentrations **(V(MOCA)_[inhibitor]_)** were divided by MOCA hydrolysis reaction rate in the presence of 0.1%DMSO (**V(MOCA)_[0.1% DMSO]_**). Inhibitory potency (%) = 100%−(V(MOCA)_[inhibitor]_/V(MOCA)_[0.1% DMSO]_)×100%.

By using Ro 32-7315 on PBMs we noticed that this specific TACE inhibitor caused significant inhibition of TACE-specific ABZ hydrolysis (almost 80%) and only a slight inhibition of MOCA hydrolysis (Figure 6). In contrast, TQ did not display any effect on TACE-specific ABZ hydrolysis on PBMs, while Celastrol induced moderate inhibition ∼25%) (Figure 6). However, both TQ and Celastrol displayed inhibitory activities on MOCA hydrolysis by PBMs (respectively ∼35% and 50%). These results were supported by those obtained in THP-1 cells, showing a significant inhibition of the MOCA hydrolysis with only minimal inhibition of ABZ hydrolysis by TQ and Celastrol (Figure 7). However, Ro 32-7315 developed as a selective TACE inhibitor, surprisingly blocked significantly the cleavage of MOCA but only minimally ABZ, the TACE specific substrate (Figure 7). These observations indicate that THP-1 cells exhibit a different enzymatic activity able to process m-TNF-α in physiological conditions. Despite it seems that both TQ and Celastrol are able to target this different TACE enzymatic activity, Celastrol is also able to block the genuine TACE. In addition, Ro 32-7315 presented some capacity to inhibit this activity, suggesting a protein structure similarity for both activities as previously illustrated by cell surface specific anti-TACE antibody recognition on the two cell types (figure 5).

### Recombinant human TACE activity inhibition study

**Figure 8:**
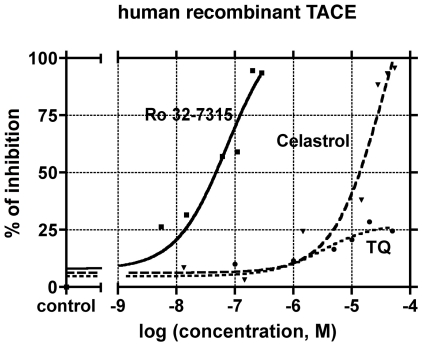
Effects of Ro 32-7315, Celastrol and TQ on rh TACE activity. 100 ng/ml of rh TACE were incubated with 5 µM of MOCA substrate in 25 mM Tris, pH 9.0, 2.5 µM ZnCl2, 0.005% Brij 35, in the presence of different compounds concentrations. The compounds were injected directly into the reaction solution every 600 s and the fluorescence intensity was monitored at λ excitation/λ emission: 325/400 nm, at room temperature. To estimate the accurate inhibitory potency of the tested compounds, the reaction rates of MOCA hydrolysis obtained for different compounds concentrations **(V(MOCA)_[inhibitor]_)** were divided by MOCA hydrolysis reaction rate with DMSO addition (**V(MOCA)_[DMSO]_**). Inhibitory potency (%) = 100%−(V(MOCA)_[inhibitor]_/V(MOCA)_[DMSO]_)×100%.

In order to validate the results of a differential enzymatic activity obtained with our cell-based fluorogenic peptide cleavage assays, human recombinant TACE was used to directly measure the effects of TQ and Celastrol on enzyme activity. Our data confirmed the strong anti-TACE potential of Ro 32-7315 (Figure 8). As formerly stated, Celastrol was able to inhibit TACE, however only at high concentrations. For TQ we obtained only a discrete rhTACE activity inhibition, even for the highest concentrations, so that the inhibition profile we obtained appears comparable to the one obtained for human monocytes when tested with the TACE specific ABZ substrate (see Figure 7 and 8). However, it is important to consider that in these latter experiments on a soluble rhTACE protein, one main actor was missing *i.e.* the influence of the cell membrane composition.

## Discussion

While inhibition of TNF-α secretion by Ro 32-7315 is attributed to blockade of TACE catalytic activity, there are no such reports for other biologically active and potentially clinically-attractive molecules (or their derivatives) such as Celastrol or TQ. It is well documented that anti-TNF-α activity of both Celastrol and TQ results principally directly from their inhibitory activity on NF-κB, a transcription factor strongly involved in induction of TNF-α production [25], [26], [34]. In order to study whether Celastrol and TQ could, as Ro 32-7315, influence TACE catalytic activity, we performed flow cytometry analysis of membrane-anchored TNF-α expression. When secretion of TNF-α increases, TNF-α on the cell surface decreases, in particular during LPS stimulation so that the presence of specific proteases inhibitors prevents TNF-α release from the cell membrane, potentially resulting in its retention and accumulation on cell surface. However, other authors such as Gearing *et al.*, reported that TNF-α secretion inhibition by metalloproteinase inhibitors does not result in membrane-bound TNF-α accumulation on *in vitro* human leukocytes cultures [10]. Additionally, Decoster *et al.* demonstrated that non-secretable, biologically active mutant of m-TNF-α expressed in L929 cells did not reveal an increased numbers of TNF-α molecules on the cell membrane, despite the fact that these molecules are not longer cleaved. The proposed explanation was that a homeostatic feedback mechanism conducted to an m-TNF-α rapid recycling and degradation inside the cells [35]. In opposition to previous authors, Shurety *et al.* showed that the metalloprotease inhibitor BB-3103 known to inhibit TACE, causes retention of m-TNF-α on LPS-stimulated RAW264 cells and this for relatively long periods, up to 16 hours [6]. In our hands and in accordance with Shutery et al. report, the selective TACE inhibitor Ro 32-7315 significantly increased the number of cells bearing high m-TNF-α levels in LPS activated THP-1 cells as well as in PBMC. In the absence of any TACE inhibitor, an increase in surface-associated TNF-α could not be observed after LPS activation, even when cells were treated by Rolipram, a non-protease inhibitor of TNF-α secretion (data not shown). These observations strongly indicate that a rapid processing and release of TNF-α to the extracellular compartment appears following LPS stimulation, and that a TACE inhibitor can effectively prevent this event resulting in a membrane anchored TNF-α accumulation. To support this concept, we provide confocal microscopic pictures showing a strong cell surface immuno-reactivity after Ro 32-7315 treatment in both THP-1 cells and human monocytes. Visualization of membrane-anchored TNF-α reveals its punctuated distribution at cell surface, either cells are being or not treated by TACE inhibitors. The characteristics of this distribution strongly suggest an association with the cholesterol-rich membrane micro-domains (lipid rafts) localization of m-TNF-α, and might play an important role in TNF-α secretion. In fact, recent finding demonstrated that TNF-α mature form is present in this cell membrane compartment and that inhibition of metalloproteases increases TNF-α proportion in those parts of the cell [31]. In addition we demonstrated the presence of TACE on both cell types, THP-1 and PBMCs, confirming that the observed TNF-α retention can really be attributed to TACE inhibition. While in our hands Celastrol and TQ showed to produce an important m-TNF-α increase expression in PBMC and THP-1 cells after LPS stimulation, well correlated with a significant decrease in s-TNF-α, we could suggest that both compounds are able to block TACE shedding activity. Subsequently, we studied TACE catalytic activity developing a cell-based fluorimetric assay of real time monitoring of enzymatic activity. The assay is based on fluorescence-quenched peptides as a substrate encompassing the cleavage sequence of TNF-α as recognized by TACE. Enzymatic hydrolysis of the peptide leads to an increase in fluorescence intensity, proportional to the amount of cleaved peptide. First we used the broad-spectrum MMP substrate (MOCAc-Lys-Pro-Leu-Gly-Leu-Dap(Dnp)-Alz-Arg-NH_2_, MOCA), particularly sensitive for collagenase (MMP-1,-8,-13) and TACE, and in a lesser extend for ADAM10. Although this substrate is not based exactly on a pro-TNF-α amino acid sequence, Neumann et al. reported its kcat/Km value as much higher than those of the standard TACE substrates mimicking the physiological cleavage site of TNF-α [36]. TACE does not possess strict crucial specificity for the A-V scissile bond cleaving efficiently the others [37]. Thus, strengthen by good water solubility, use of this substrate in our shedding assays allowed us a sensitive detection of TACE activities on in situ cell surfaces. Results obtained with the MOCA substrate were then compared with those obtained with a second, this time highly specific, TACE substrate: Abz-Leu-Ala-Gln-Ala-Val-Arg-Ser-Ser-Ser-Arg-Dap(Dnp)-NH_2_ (ABZ) [37]. This latter is based on the amino-acid sequence strictly identical to the scissile region of the genuine pro-TNF-α allowing a highly specific determination of TACE shedding activity. Hydrolysis study of MOCA and ABZ substrates by non-activated THP-1 cells and human monocytes showed that both cell types are able to cleave each substrate (data not shown), clearly indicating the presence of a constitutive enzymatic activity on the cells surface explaining this result. In addition, we showed that TACE expression on both cell types is independent on LPS stimulation, suggesting a constitutive level of TACE activity sufficient for TNF-α release. This is consistent with the findings of Doedens *et al.* reporting that THP-1 cells LPS stimulation has no effect on TACE localization and expression [33]. One of the possible explanations of this observation may be that LPS first induces TNF-α synthesis before regulating constitutive TACE activity [33] (*suggesting that* the molecular structure of TACE could be changed or degraded [14]). However, it should be taken into consideration, that TACE surface expression might not be an adequate indicator of its cellular activity, especially that a TACE-mediated ectodomain shedding may occur in an intracellular compartment in addition to the cell surface. Furthermore, it has also been shown that TACE is predominantly localized at a perinuclear compartment similar to that described for TNF-α [38]. To date there is no report about the proportion of shedding occurring at the cell surface compared to intracellular compartments. However, noting that the membrane-anchored protein form of TACE represents the majority of its mature form, it can be suggested that at least the majority of the shedding takes place on cell surface. Studying further the effects of the tested potential TACE inhibitors on MOCA and ABZ hydrolysis by both THP-1 cells and human monocytes, we showed that Ro 32-7315, Celastrol and TQ all induced a significant reduction of MOCA peptide hydrolysis by THP-1 cells, although none of the inhibitors were able to completely abrogate the MOCA cleavage. Surprisingly, none of them were able to strongly inhibit ABZ hydrolysis as obtained for MOCA. Using such a highly specific TACE substrate (ABZ) and a highly selective TACE inhibitor (Ro 32-7315) we expected to gain complete abrogation of ABZ hydrolysis, indeed we obtained only 20% abrogation. Consequently, the fact that about 80% of constitutive TACE activity remains unchanged after Ro 32-7315 treatment, might suggest that in physiological conditions, TACE could occur in a conformational different state then following LPS activation, less accessible to this inhibitor. A similar consideration can be hypothesized for Celastrol and TQ, inhibiting hydrolysis of ABZ substrate for about 40% (Celastrol) or 20% (TQ) and MOCA substrate for about 85% or 58%, respectively. In support came our observations on human monocytes. In these cells, Ro 32-7315 induced almost 80% ABZ hydrolysis inhibition, and this with only a little inhibition of MOCA hydrolysis, confirming its high anti-TACE potential. For Celastrol and TQ we obtained a similar profile in THP-1 cells, although the capacity of both compounds to inhibit MOCA and ABZ substratehydrolysis was much less pronounced. These results indicate that unlike Ro 32-7315, Celastrol and TQ trigger a better TACE inhibition on activated THP-1 cells. To amplify our findings, we studied the effects of all three compounds on human recombinant TACE activity. As expected, our results for rhTACE confirm a strong inhibitory potency of Ro 32-7315. Celastrol, but not TQ, can also efficiently inhibit TACE activity, however only at much higher concentrations.

In summary we demonstrate here that:

cell surface TACE protein is present on THP-1 and PBMC in a rate independent of cellular activation,
Ro 32-7315, Celastrol and TQ inhibit TACE activity as illustrated by important increase in m-TNF-α expression as well as real time monitoring of enzymatic activity,
independently of the cell type, THP-1 or monocyte, as for recombinant TACE protein, hydrolysis of the broad-spectrum MMP substrate MOCA is inhibited more or less but in an equivalent manner respectively by Ro 32-7315, Celastrol and TQ.
on the contrary, hydrolysis of the highly specific TACE substrate Abz if strongly inhibited by Ro 32-7315 on monocyte, for THP-1 cells only a 20% inhibition is obtained.

Our data suggest that in human THP-1 cells, TACE activity is catalytically changed after LPS activation, compared to human monocytes where TACE appears less sensitive to the presence or stimulation by LPS as shown by the differential inhibitory activities of Ro 32-7315, Celastrol or TQ. Thus the THP-1 cell line might not be a good cell line to screen chemical libraries in the quest of new TACE inhibitors.

## Materials and Methods

### Reagents and compounds

Biotin mouse anti-human TNF-α antibody, purified anti-human TNF-α (capture) antibody, recombinant humanTNF-α and CellFIX® were purchased from BD Biosciences Pharmingen (Le Pont de Claix, France). Phosphate buffer saline (PBS) Dulbecco w/o Ca^2+^, Mg^2+^ (PBS Instamed) was from Biochrom AG (VWR, Fontenay-sous-Bois, France). Fetal Bovin Serum (FBS) was obtained from Lonza BioWhittaker (Fisher Bioblock Scientific, Illkirch, France). Brj 35 detergent, 30% aqueous solution was from Calbiochem/Merck (VWR, Fontenay-sous-Bois, France). Penicillin-streptomycin was purchased from Cambrex Bio Science (St Beauzire, France). 96 wells culture plates was from Corning International (Avon, France). L-glutamine was obtained from GIBCO (Invitrogen, Cergy Pontoise, France). 24-wells tissue culture treated plates, 25 and 75 cm^2^ polystyrene sterile flask, and15 or 50 ml polypropylene tube were from Greiner bio-one (Courtaboeuf, France). Guava Nexin Assay kit, Guava ViaCount kit and 7-AAD were purchased from Guava Technologies (CA, USA). Tris(hydroxymethyl)-aminomethan was from Merck (Lyon, France). 1,4 ml U-tubes and Nunc maxisorp plates were from Micronic (Dominique Dutscher, Brumath, France). FRET substrates: Abz-Leu-Ala-Gln-Ala-Val-Arg-Ser-Ser-Ser-Arg-Dap(Dnp)-NH2) and MOCAc-Lys-Pro-Leu-Gly-Leu-Dap(Dnp)-Ala-Arg-NH2 were purchased from Peptides International (Louisville, KY, USA). Monoclonal anti-human extracellular TNF-α-Fluorescein, monoclonal anti-human TACE-Phycoerythrin and Human recombinant TACE were obtained from R&D Systems Europe (Lille, France). Dimethyl Sulfoxide (DMSO), Hank's balanced salt solution (HBSS), Histopaque-1077, Lipopolisaccharide (LPS) from Salmonella abortus equi, RPMI 1640, SigmaFast OPD (O-phenylenediamine dihydrochloride), Thimerosal (Mercury-[(o-carboxyphenyl)thio]ethyl sodium salt), Tween 20 and Zinc chloride ZnCl_2_ were obtained through Sigma-Aldrich (Saint-Quentin Fallavier, France). Vectashield® Hard Set™ was from Vector Laboratories (CliniSciences, Montrouge, France). Streptavidin-Horseradish peroxidase (HRP) was from Zymed (Clinisciences, Montrouge, France). Ro 32-7315 was a generous gift of Dr. Keith Walker (Roche, Palo Alto, CA USA), as Celastrol by Pr. AC Allison (Alavita Pharmaceuticals, Mountain View, CA USA).

### Cells, cell cultures and cells incubations

The human monocytic cell line THP-1 (from the American Type Culture Collection, ATCC) was routinely maintained in RPMI 1640 culture medium supplemented with 10% (v/v) heat inactivated FBS and penicilin-streptomycin (100 IU/ml and 1 µg/ml).

The human peripheral blood monocytes, purchased at the “Etablissement Français du Sang-Alsace” (EFS, Strasbourg, France), were a generous gift of Professor Sylvie Fournel (Immunologie et Chimie Thérapeutiques UPR 9021 CNRS, IBMC, Strasbourg, France). The cells were obtained by elutriation from healthy donors with no clinical history. The used cell suspension (in RPMI 1640 culture medium supplemented with 10% (v/v) heat inactivated FBS and penicilin-streptomycin (100 IU/ml and 1 µg/ml)) contained 70% of monocytes and 10% of granulocytes.

For human blood mononuclear cells (PBMC) studies, healthy donors with no clinical history were selected. Written consent from each donor was obtained following the recommendations of the “Comité de Protection des Personnes (CPP) Nord Ouest II” which gave his agreement the 12/07/2006 followed by the agreement of the “Direction Générale de la Santé” the 01/21/2007 (protocol #2006-A00319-42), and the work carried out according to international guidelines.

PBMCs were separated in a single-step density centrifugation technique, as previously described [39]. Briefly, peripheral blood diluted in Ca^2+^ and Mg^2+^ free (CMF) HBSS (CMF-HBSS) was layered over Histopaque-1077 and centrifuged for 30 minutes at 400 g (20°C). Cells harvested from the interface were washed 3 times in CMF-HBSS and re-suspended in a culture medium consisting in RPMI 1640 supplemented with 10% (v/v) heat inactivated FBS and penicillin-streptomycin (100 IU/ml and 1 µg/ml). THP-1 cells and human monocytes were incubated in 96 wells culture plates for 4 hours, human PBMCs for 24 hours, at 37°C in a humidified 5%CO_2_/95% air atmosphere in presence of increasing doses of the tested compounds. The final concentration of cells was 5×10^5^/ml in a final volume of 250 µl per well. Lipopolysaccharide (LPS) from *Salmonella abortus equi* was used to induce TNF-α secretion by cells (1 µg/ml for THP-1 cells and human monocytes and 5 µg/ml for PBMCs). Optimal LPS (from *Salmonella abortus equi*) concentrations have been established in previous experiments as optimal LPS concentrations vary from one bacterial species origin to another, and differ also considering the studies which would be performed (data not shown). Compounds, dissolved in DMSO, were diluted in culture medium and added to the cells cultures with a DMSO final concentration never exceeding 0.1% (v/v). Control samples always contained the same amount of DMSO (without drug) to exclude any interference of DMSO in cell responses.

After the incubation period, the supernatants were removed and stored at −20°C prior to ELISA assay.

### Compound's toxicity measurements

The Guava Nexin Assay was used for discriminating viable, necrotic and apoptotic cells by the means of Annexin V-PE and 7-AAD labeling. Annexin V-PE detects phosphatidylserin (PS) on external membrane of early and late stage apoptotic cells, whereas 7-AAD, a non permeant dye, detects late stage apoptotic cells and dead cells. Prior to the Guava Nexin assay, LPS activated PBMC or THP-1 cells were incubated in 96 wells culture plates for 24 hours and 4 hours respectively, at 37°C in humidified 5% CO2 (95% air atmosphere) in the presence or absence of the tested compounds. The supernatants were then removed and the cells were resuspended in 200 µl of fresh culture medium and stained with 20 µl of Annexin V-PE and 7-AAD mixture (1/1 V/V in fresh culture medium). After 20 minutes of incubation (in the dark at room temperature) the samples were analyzed on the Guava EasyCyte Plus System (Guava Technologies) equipped with a 488 nm excitation laser and four emission filters at 520/40, 585/42, 675/30 and 780/30 nm. The samples were analyzed using Guava Express® Plus software. Granulation, size and fluorescence intensity were recorded for 5,000 cells.

### Soluble TNF-α detection and quantification by ELISA

Quantitative evaluation of secreted TNF-α in culture supernatants was achieved by a two-site Enzyme-Linked Immunosorbent Assay (ELISA), as previously described [40]. Polyvinyl chloride plates were coated with 100 µl per well of purified anti-human TNF-α capture antibodies (2 µg/ml) and incubated overnight at 4°C. After washing and saturation steps, 50 µl of standard or samples were added to 50 µl of biotinylated monoclonal antibody (1 µg/ml) for 2 hours at room temperature. Following washing steps, 100 µl of a peroxidase streptavidin dilution (1/2000 in PBS) were added (45 minutes at room temperature). A colorimetric reaction using 0-phenylenediamine dihydrochloride (OPD) as peroxidase substrate was performed ensuing three washing steps. The optical density (OD) of each well was determined at 450 nm using Versamax microplate reader linked to the SoftMax Pro software (Molecular Devices). TNF-α concentrations (pg/ml) of unknown samples were computed by interpolation with a standard curve run on each plate using four parameters logistics analysis.

### Immunofluorescence staining for membrane bound TNF-α and TACE detection

The cells to be stained were incubated in 24 wells culture plate for 4 hours (for THP-1 cells and monocytes) or 24 hours (PBMC), at 37°C in humidified 5% CO_2_ (95% air atmosphere). The final cellular concentration was 5×10^5^ cells/ml for 1 ml per well. The cells were activated with LPS: 1 µg/ml for THP-1 cells and monocytes or 5 µg/ml for PBMC. For TNF-α detection cells were incubated in the presence of the tested compounds or 0.1% DMSO (control samples).

Following incubation period, the cells were harvested and washed in CMF-HBSS supplemented with 0.5% FBS. Prior to staining, cells were Fc-blocked by treatment with 1 µg of human IgG for 15 minutes at room temperature. Each sample was then placed on ice and finally incubated 30 minutes with monoclonal anti-human extracellular TNF-α or monoclonal anti-human TACE. Cells were then washed in CMF-HBSS supplemented with 0.5% FBS, centrifuged at 500 g for 1 minute and fixed in 500 µl of CellFix® (Becton Dickinson). The samples were analyzed by capillary flow cytometry.

### Flow cytometry analysis

All samples were run on Guava EasyCyte Plus System (Merck Millipore, Life Science division, Merck KgaA, Darmstadt, Germany) equipped with a 488 nm excitation laser and four emission filters at 530/40, 585/42, 675/30 and 780/30 nm. Samples were analyzed using Guava ExpressPro software (Merck Millipore Guava Tech). A dot plot gate was used on forward and side scatter to eliminate debris. Granulation, size and fluorescence intensity were recorder for 5,000 cells per analysis.

### Image acquisition by confocal microscopy

The stained cells were washed in CMF-HBSS, then incubated with 7-AAD for 15 minutes in ice. Following one washing step, the cells were finally resuspended in CMF-HBSS supplemented with 0.5% FBS for slide preparation. To mount cells on slide, Vectashield®Hard Set™ Mounting medium was used. Image acquisition was made using an inverted confocal microscope Leica SP5 (Leica Microsystems GmBh) and the acquisition software Leica LAS AF. The numerical recording of the images and it analysis was carried out with ImageJ, a freeware created by Wayne Rasband (NIH, USA).

### Fluorometric assay for protease activity on living cells

Protease activity measurements were performed by continuous monitoring of Fluorescence Resonance Energy Transfer (FRET) substrates cleavage that is real-time increases in fluorescence intensity. Two FRET substrates (Peptides International, USA) were use: MOCAc-Lys-Pro-Leu-Gly-Leu-Dap(Dnp)-Ala-Arg-NH_2_ (MOCA, substrate for MMPs, Cathepsin D and E, ADAM10 and ADAM17/TACE, cleaved at the Gly-Leu scissile bound) and Abz-Leu-Ala-Gln-Ala-Val-Arg-Ser-Ser-Ser-Arg-Dap(Dnp)-NH_2_ (ABZ, substrate for TACE, corresponding to the human pro-TNF-α sequence surrounding the TACE specific cleavage site (Ala-Val bound)). The fluorescent signals were measured using cuvette-based spectrofluorometer DeltaRam, computed on the FeliX32 Fluorescence Analysis software 1.1 (Photon Technology International), at λ_ex_/λ_em_: 325/400 for MOCA and 320/420 for ABZ.

Prior to the fluorimetric assay, non activated cells were washed in CMF-HBSS and then resuspended in the same buffer to a final concentration of 5×10^5^ cells/ml. Cell viability was determined using Guava®ViaCount, and routinely obtained viability was more than 95%. Cells were dispensed into a plastic (UV compatible) 1-cm cuvette. For inhibitor studies, tested compound, dissolved in DMSO, or DMSO alone (control samples) were added to the cells prior to addition of substrate. Finally, MOCA or ABZ substrate was dispensed directly into the cuvette at a final concentration of 5 µM and fluorescence measurements were initiated. The final volume of the samples was always 1.5 ml and final DMSO concentration never exceeded 0.1%. All assays were carried out under agitation at room temperature. Cleavage of the substrate was monitored continuously for 600 s for each experimental point. The initial velocities (Vo) of the substrates hydrolysis were determined from the linear section of the fluorescence curves.

### Recombinant human TACE enzymatic activities

Recombinant human TACE protein corresponding to the secreted mature form of TACE with N-terminal sequence RADPDPMKNT, was obtained from R&D Systems Europe. Prior to fluorescence measurements, rhTACE was diluted in 1.5 ml of assay buffer (25 mM Tris, pH 9, 2.5 µM ZnCl_2_ and 0.005% Brij) to a final concentration of 100 ng/ml. The enzymatic reaction was initiated by addition of MOCA substrate to a final concentration of 5 µM. The tested compounds, dissolved in DMSO, or DMSO alone (control samples) were then added directly into the reaction solution every 600 s. The final titrated concentrations of the compounds ranged from 0.01 to 100 µM. All fluorescence measurements were carried out under agitation in 1-cm quartz cuvette at room temperature. The initial velocities (Vo) of the substrate hydrolysis were determined from the linear section of the fluorescence curves.

### Statistical analysis

All statistical analyses were computed with GraphPad PRISM® (Version 4.0 Software Inc., San Diego CA). Results are given as mean ± SD (standard deviation) from “n” independent experiments. The half-maximal inhibition values (IC50) for each tested compounds is determined by fitting the data to the sigmoid dose-response equation:
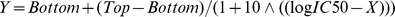
where X is the logarithm of the compound's concentration, Y the biological response, Bottom the baseline response and Top is the maximum response.

Comparison analyses are performed using paired Student's t-test or Mann-Whitney non-parametric test. Statistical significance is accepted for a value of p<0.05. For enzymatic analysis, initial velocities (V0) are computed from the linear section of the fluorescence curves of substrate hydrolysis using linear regression analysis. Km (Michaelis Menten constant) and Vmax (the maximum enzyme velocity of substrate hydrolysis) values are obtained by fitting the data to equation:
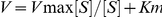
where S is the substrate concentration and V is the enzyme velocity of substrate hydrolysis.
